# Enhancing disaster response of emergency medical teams through “TEAMS 3.0” training package—Does the multidisciplinary intervention make a difference?

**DOI:** 10.3389/fpubh.2023.1150030

**Published:** 2023-04-12

**Authors:** Arielle Kaim, Moran Bodas, Nieves Amat Camacho, Kobi Peleg, Luca Ragazzoni

**Affiliations:** ^1^Israel National Center for Trauma and Emergency Medicine Research, Sheba Medical Center, The Gertner Institute for Epidemiology and Health Policy Research, Ramat-Gan, Israel; ^2^Department of Emergency and Disaster Management, Faculty of Medicine, School of Public Health, Sackler Tel Aviv University, Tel Aviv, Israel; ^3^Department of Global Public Health, Center for Research on Health Care in Disasters, Karolinska Institute, Stockholm, Sweden; ^4^CRIMEDIM-Center for Research and Training in Disaster Medicine, Humanitarian Aid and Global Health, Università del Piemonte Orientale, Novara, Italy

**Keywords:** emergency medical teams, intervention, education, training program, self-efficacy, teamwork, quality of training

## Abstract

**Background:**

In the aftermath of disasters, Emergency Medical Teams (EMTs) are dispatched to help local rescue efforts. Although EMTs are recognized to be a critical component of the global health workforce, concerns have emerged over their functioning and effectiveness. For example, lack of cooperation and coordination between different EMTs has been a longstanding issue, resulting in fragmented disaster management.

**Methods:**

To enhance the provision of EMT’s field teamwork, the Training for Emergency Medical Teams and European Medical Corps (TEAMS) project was established, and later further updated with novel scenarios and exercises (i.e., adapting EMT operations to a sudden disaster; becoming a modular team; reflecting on ethical dilemmas) in the complementary “TEAMS 3.0” project where a more comprehensive training package was developed. The aim of this study was to assess the effectiveness and quality of the TEAMS 3.0 training package in four training programs in Portugal, Germany, Norway, and Turkey. Participants completed a set of questionnaires designed to assess self-efficacy, teamwork, and quality of training.

**Results:**

The results from all the trainings suggest an improvement for both teams’ self-efficacy and teamwork. The mean score among all the participants (*N* = 100) for both the self-efficacy scale and teamwork scale was 3.217 (±0.223) prior to training and 3.484 (±0.217) following the training, and 2.512 (±1.313) prior to training and 3.281 (±0.864), respectfully, with statistically significant differences according to Wilcoxon paired samples test (*p* < 0.05). The quality of training is regarded as high and deemed as an appropriate tool package for addressing the objectives of the project and the perceived needs of EMT disaster deployment.

**Conclusion:**

Thus far, the TEAMS 3.0 project has demonstrated to be effective in promoting EMT teamwork capacities.

## 1. Introduction

Despite commitments and investment to better prepare for and mitigate disasters, build resilience, and address climate change, risk creation continues to outstrip risk reduction. Disasters and the underlying vulnerabilities that drive risk are increasing, with future such challenges faced only expected to magnify (1). In the event of a disaster, individuals and infrastructures are often put at unforeseen risk (2). In such contexts, the international community offers various forms of assistance, including the deployment of emergency medical teams (EMTs)—defined by the World Health Organization (WHO) as *“*groups of health professionals and supporting staff outside their country of origin, aiming to provide health care specifically to disaster affected populations (3).

The EMT initiative evolved in 2010, under the umbrella of the WHO with the overall objective to save lives and preserve health by enhancing surge capacity to host countries through the provision of basic and/or advanced care. EMTs include both governmental (civilian and military) as well as non-governmental teams, which as of 2022, 37 global teams have undergone complete classification (4). Synchronous to WHO efforts, in 2016 the European Union (EU) launched the European Medical Corps (EMCs) to help mobilize medical and public health teams and equipment for emergencies inside and outside the EU (5).

Despite playing a critical role in the global health workforce, previous EMT and EMC deployments have demonstrated shortcomings and concerns over their overall efficacy and functioning. Concerns have included poor preparedness and equipping to the disaster setting, failure to coordinate with response authorities and additional relief agencies, and lack of previous experience in disaster response (6). The devastating Haiti earthquake of 2010 highlighted to the global response community the adverse implications of relief that is inappropriate, uncoordinated, unsustainable, and unprepared, albeit nobly intentioned (7). Lessons have indicated that the provision of poor aid have repercussions for both the relief efforts of the affected population and may be traced among humanitarian aid workers themselves. While often overlooked, a longstanding consequence of unprepared deployment and lack of experience in the field of disaster response are the documented post-disaster rates of anxiety, depression, and trauma symptoms among humanitarian aid workers, which further diminishes the aptitude of responders to aid the community they initially intend to serve (8). To improve the quality, preparedness, and professionalism of deployed teams, it has been established that standardized guidelines and a coherent methodology to training is key.

In recognition of these elements, global health agencies have published various guidelines to improve the quality of the medical response by EMTs—most importantly the WHO’s ‘*Classification and minimum standards for foreign medical teams in sudden onset disasters’* (9). In addition, multiple organizations and academic bodies have developed educational programs for disaster and emergency response; with a significant variation in robustness, curriculum and quality (10–14). The Training for Emergency Medical Teams and European Medical Corps (TEAMS) project is one such training program focused on operational team training for EMCs/EMTs established with the goal of enhancing the efficient provision of field team work. The projects’ objective was to develop, pilot and assess a standardized, validated and cost-effective training package which was adaptable to different types of EMCs/EMTs, and was sustainable within low income countries and resource poor settings (15). The project successfully demonstrated preliminary data from two pilot training programs in Germany and Turkey in 2018, which demonstrated the effective development of a high- quality training package comprising of a set of 8 innovative blended-learning teaching materials and simulation-based exercises (see Table 1).

**Table 1:** Outline of TEAMS original training package consisting of 8 exercises.

#	Exercise title	Type of exercise	Phase of the humanitarian mission	Exercise scope	Learning objectives
1	Preparing for deployment	Tabletop exercise	Pre-deployment	This exercise simulates the first meeting of a group of EMT members assigned to deploy in response to an earthquake in a fictitious country. Before heading to the field, the team members introduce to each other, get information about the mission and understand what their roles will be once on the field. They will also have to work together on different preparatory tasks for the imminent deployment.	To effectively manage the information received before deployment To understand the different EMT staff roles within the team To work collaboratively for the preparation of the EMT deployment
2	Arriving and setting up	Functional exercise	Arrival and set up	This exercise simulates the arrival and set up of the EMT in the field. On arrival participants will need to meet relevant authorities and organizations managing the response to the earthquake, obtain important information, and get registered to work as an EMT in the country.	To be aware of the communication and registration procedures on arrival in the disaster area To build up the field hospital in the target area To get familiar with the field equipment and logistics
3	Setting priorities	Functional exercise	Operational	During this exercise the EMT members will be confronted with patients in very critical conditions and a set of resources to treat these patients. The team will have to decide how to allocate the available resources in order to save the highest number of patients. A role player will also intervene during the exercise, taking the role of a father whose child is admitted within the EMT facility in a critical state.	To manage situations involving difficult ethical decisions To navigate between needs and resources in a critical situation To maximize the response to a critical event with the available resources and the network around
4	Managing operational information	Tabletop exercise	Operational	In this exercise team members will receive different sources of information related to EMT activities that they will read and consider to plan for their activities in the upcoming days. This planning will be shared with the EMT HQ office in a situation report. The team will also have to report their activities to the EMT Coordination Cell (EMTCC) using the Minimum Data Set (MDS) forms.	To recognize the main tools for EMT data collection and reporting To correctly analyze and interpret data related to EMT activities To report EMT data following the established channels To deal with emerging situations while performing other routine tasks To work collaboratively during data collection and reporting tasks
5	Responding to a mass casualty incident	Functional exercise	Operational	In this exercise a Mass Casualty Incident (MCI) will be simulated, following an aftershock. The whole team will have to organize to deal with the high number of casualties arriving at the EMT facility, while constantly communicating with the EMTCC and other partners in the area.	To effectively communicate with the EMTCC for situation awareness and coordination of a MCI To appropriately organize as a team and manage a MCI
6	Adapting practice to context	Functional exercise	Operational	During this exercise EMT members will have to develop or adapt an available Standard Operating Procedure (SOP) for the management of dead bodies in the local context. Once this is ready they will be confronted with a case of a boy who arrives at the EMT facility and dies shortly after. The team will have to consider the circumstances in which the child was brought in the facility and interact pertinently with the family.	To adapt EMT procedures to the local context To manage a clinical emergency case of an unaccompanied minor To show empathy and responsibility when handling sensitive cases To understand the position of an EMT during disaster response and work collaboratively with other partners
7	Planning the exit	Tabletop exercise	Exit	In this exercise, participants will prepare for the EMT exit by planning for the handover of medical activities, logistics, dealing with the local staff and the local community, the management of medical records and possible donations to the local facilities.	To identify the main actions required for the EMT exit To understand the importance of adapting the exit strategy to the local context To effectively deal with the media during emergencies To work collaboratively toward the exit
8	Dealing with security threats	Functional exercise	Exit	The module presents a commonly encountered case scenario in humanitarian settings and stresses the importance of both proper planning before undertaking overland road travels and adequate team/individual behavior when crossing checkpoints.	To understand the reasons of the road movement To plan the trip in order to reduce vulnerability during the overland road travel To demonstrate good skills in the utilization of satellite-based navigation and other communication devices To demonstrate good knowledge of the basic behavioral tips when crossing a checkpoint To demonstrate good communication skills To demonstrate good negotiation skills

CC. Disaster medicine and public health preparedness (15).

The TEAMS 2.O project was the simultaneous and overlapping project to that aimed to create a Training of Trainers’ (ToT) program to train novice and inexperienced team leaders or training managers of EMCs/EMTs organizations on how to effectively use the TEAMS training package.

The TEAMS 3.0 project is the continuation and complementary project to the TEAMS 1.0 project, which aims to expand and revise the training package given the unceasing evolution of both the EMC and EMT initiatives. The previous TEAMS package solely addressed natural disasters (earthquake), where scenarios and exercises were lacking to train capacities to respond to outbreaks, man-made disasters and other possible cross-border risks. Additionally, only two EMT organizations had previously officially delivered the entire TEAMS training package.

Therefore, the study aimed to evaluate the follow-up TEAMS 3.0 training package involving developed new scenarios and exercises as well as new partners based on two aspects: (a) assessment of the effectiveness of the training package to increase self-efficacy and team-work of EMT participants, and (b) assessment of the quality of the training program organized.

## 2. Methods

### 2.1. Study design

This study examined the change in 3 major constructs, i.e., team work, perceived self-efficacy, and perceived quality of training, among participants of the TEAMS Training (see following). The comparison was conducted for each participant responding to questionnaires administered before and immediately after the training.

The TEAMS 3.0 Training Package and Platform was designed to support the development and improvement of EMTs’ teamwork. Through a series of three additional exercises added to the original training package (of eight exercises) which widens the scope of the package to create a more comprehensive package for personnel to train scenarios likely to be met on the field, while focusing on the importance of teamwork in achieving their goals.

The TEAMS Training Package is comprised now of a set of eleven innovative blended-learning teaching materials and simulation-based exercises. Each exercise is a complete stand-alone module consisting of a concept note, learning objectives sheet, debriefing tool, and a variety of supplementary documents aimed at facilitating the exercise, such as injects, annexes, reading materials and gaming accessories.

The three additional exercises developed for the purpose of the TEAMS 3.0 training package include the adaptation of EMT operation to a sudden disaster (sudden outbreak or chemical incident); becoming of a modular team; and reflecting on ethical dilemmas (see Table 2).

**Table 2:** Outline of three new exercises added to the original training package (See above).

Exercise #	Title	Phase of disaster response	Exercise scope	Learning objectives
A	Adapting EMT operations to a (A) sudden outbreak/(B) chemical incident	Operational Response	A. When the team has already started their activities at the EMT facility in Montyland, they will receive information about the possible outbreak of severe acute respiratory infection (SARI) in the area. With the initial data provided and others they can collect, they will have to assess emerging needs and prepare actions to install protective measures for the staff and the patients, change workflows, establish referral systems and manage suspect cases arriving at the facility. B. When the team has already started their activities at the EMT facility in Montyland, they will receive information about cluster of cases suspected to be chemical poisoning followed shortly by an explosion of chemical factory in the area. With the initial data provided and others they can collect, they will have to assess emerging needs and prepare actions to install protective measures for the staff and the patients, change workflows, establish referral systems, and manage suspect cases arriving at the facility.	A: To perform an initial needs assessment after the suspicion of an infectious disease outbreak in the area To plan necessary actions for the potential arrival of suspected cases to the EMT facility To carry out the planned measures on-site (EMT temporary facility) B: To perform an initial needs assessment after the suspicion of a chemical incident in the area To plan necessary actions for the potential arrival of suspected cases to the EMT facility To carry out the planned measures on-site (EMT field hospital)
B	Becoming a modular team	Operational Response	Since the work at the EMT in the facility has significantly decreased, the team will be unexpectedly requested to undertake different tasks for which they will need to split into smaller groups. Team members will need to arrange the groups and prepare to deploy in different areas for given assignments. Once deployed to new posts, they will have to deal with upcoming challenges.	To effectively prepare for unexpected deployments To provide respectful and targeted support to local health facilities To reflect on possible upcoming challenges related to operational work
C	Reflecting on ethical dilemmas	Operational Response	Team members will be presented with different cases to reflect on, first individually and then as a group, and they will have to decide among different options what they think is the best way to act.	To reflect on ethical dilemmas possibly presenting during deployments To develop a constructive team dynamic to discuss ethical dilemmas and make decisions

### 2.2. Training of trainers course

In anticipation of the TEAMS 3.0 trainings, a Training of Trainers (ToT) Course was held in a virtual format resulting from the COVID-19 pandemic on 22–24 of November 2021. The ToT course hosted twelve trainees, training to become TEAMS trainers in the Portuguese, Norwegian, German, and Turkish trainings, which were held later throughout 2022.

The in-training trainers analyzed the eleven exercises individually to explore means to implement their purpose and intended contribution to the training EMT. They also discussed in depth gaps, challenges, and expected hardships in implementing the exercises and means to overcome them. In addition, they participated in several lectures designed to provide tools and insights to the work of a trainer, as well as to tap into experts’ experience from the field.

### 2.3. Trainings

The trainers who underwent the ToT course eventually trained their own respective EMT trainees. These trainings were recently frontally held in all four countries (Portugal, Germany, Norway and Turkey) in the context of the TEAMS 3.0 Project. Two of the trainings (Portugal and Turkey) conducted a full training of all eleven exercises, while Germany conducted, the full training however in a modular format (where only the three new exercises were evaluated) (see Table 2), and Norway trained only the three new exercises. The training in Germany took place between 6-8th of May, 2022 and was conducted by Johanniter International Assistance, a WHO-certified Type 1 Fixed EMT. The training conducted in Portugal by Instituto Nacional De Emergência Médica- INEM, a WHO-certified Type 1 Fixed EMT took place in Lisbon, from the 26th–29th of May, 2022. The training in Turkey took place in Istanbul (Turkey) between 3rd and 6th of August 2022 and was conducted by Istanbul Medeniyet University, which overlooks the activities of a Type 2 EMT. The training in Norway was conducted from the 5th to 7th of September, 2022 in Starum, Norway by the Norwegian Directorate of Health.

### 2.4. Population and sample

Overall, 32 Portuguese, 18 German, 11 Norwegian, and 39 Turkish participants underwent the TEAMS 3.0 training and were included in the final analysis. In each training, there were three local trainers and the remainder of the participants were trainees (physicians, nurses, paramedics, emergency medical technicians, psychologists, logisticians, and coordinators.).There was a 100% response rate among all trainees. Among trainers, 10 out of 12 has completed the entire duration of the delivered training package, and thus two were unable to complete the post-training evaluation (and thus not included in the final dataset). All participants in the training and subsequent evaluations were EMT employees/volunteers who are expected to be deployed to disaster-affected areas upon need. All participants were invited to be included in the evaluation’s sample. Informed consent was obtained from the participants.

### 2.5. Variables

The TEAMS 3.0 project utilized an evaluation scheme that focused on three components: (a) Perceived teamwork, (b) Perceived self-efficacy, and (c) Training quality. The evaluation scheme was agreed upon by mutual consent and agreement of all the consortium partners. Nevertheless, given that the ToT component included within the TEAMS 3.0 Project is aimed at developing competencies and skills on an individual level the tools were adapted accordingly.

The evaluation of the TEAMS 3.0 training focused on three main constructs: *General Self-efficacy* – this index measures individual perceptions of one’s capabilities to galvanize motivation, cognitive resources, and courses of action needed to meet given situational demands; *Teamwork* – this index measures individual perceptions of leadership, team dynamics, situation awareness, and effective task management; *Quality of Training* – this index measures individual perceptions of the overall efficacy, appropriateness, and contribution to the team.

### 2.6. Tools

Assessment of the selected variables was conducted using validated and/or original measurement tools created or adapted for the purpose of this evaluation (see complete tools in Annex 1): (a) Self-efficacy of the team was assessed using an adapted version of a scale developed by Schwarzer and Jerusalem (1995) (16); (b) Teamwork was assessed using the validated tool “Team Emergency Assessment Measure”; (c) Quality of training was assessed using a questionnaire specifically designed for the purpose of this evaluation.

All assessment tools were based on Likert-scale measurement. Self-efficacy was assessed using a Likert-scale ranging from 1 (“Not at all true”) to 4 (“Exactly true”). Teamwork was assessed with a Likert-scale ranging from 0 (Never/hardly ever) to 4 (Always/Nearly always), and Quality of Training was assessed using a Likert-scale ranging from 1 (Strongly disagree) to 5 (Strongly Agree). See Table 1 for summary of tools and evaluation methodology. See all tools in Annex 1.

In the trainings, the original English versions of the questionnaires were used (with the exception of Turkey). In Turkey, all questionnaires were translated into Turkish and were administered in Turkish. Prior to that, validation of translation accuracy was conducted by translating the Turkish version back to English by an independent translator and compared the result to the original.

### 2.7. Procedure

Participants were informed during the first day of the training week about the evaluation process and its purpose. Informed consent was requested from participants willing to partake in the evaluation process. The data was collected anonymously, following approval of the Ethics Committee of Sheba Medical Center (number SMC-9777-22 from October 2, 2022). Subsequently, participants were asked to complete the first round of data collection by completing the Self-efficacy and Teamwork questionnaire online *via* Google Forms platform. The information collected at this stage is considered the “pre-training” data. Upon the completion of the last day of training, participants were asked to re-take the Self-efficacy and Teamwork questionnaires, as well as to complete the Quality of Training questionnaire, once again *via* the Google Forms platform. The information collected at this stage is considered the “post-training” data. For the sake of cross-referencing the responses, participants were asked to indicate a short, designated ID tag on their questionnaire in a manner that will allow matching of the data without compromising their anonymity (see Table 3).

**Table 3:** Summary of evaluation methodology and assessment tools used.

Assessment parameter	Participants	Proposed tool	Administration times
General self-efficacy	Trainees Trainers	Questionnaire developed by Schwarzer and Jerusalem (1995) (16)	Before and after the training
Teamwork	Trainees Trainers	Questionnaire based on the validated tool “Team Emergency Assessment Measure”	Before and after the training
Quality of training	Trainees Trainers	Original questionnaire (Trainees and trainers provided their perception of training package quality in separate questionnaires)	After the training

### 2.8. Statistical analysis

The statistical analysis of the results was performed using IBM’s SPSS Version 27. The analysis included both descriptive and analytical methods, and the statistical tests were chosen according to variables distribution. Prior to analysis, indices were generated, and their reliability was assessed using Cronbach’s Alpha.

Given the small sample size, non-parametric tests were used. Spearman correlation test (with multiple comparison correction) was used to examine correlations between continuous variables. Mann–Whitney U and Wilcoxon tests were used to compare means of independent and paired categorical variables, respectively. In all statistical analyses performed, a value of *p* of 0.05 or less was deemed as statistically significant. Missing data was excluded.

## 3. Results

Of the total sample participants (37%) were female and among trainers, 3 out of the 10. The mean participant age was 40.36 (±9.31). The average participant ages in Germany, Portugal, Turkey and Norway were 40.65 (±12.06), 41.62 (±6.82), 41.62 (±7.26), and 50.08 (±6.76), respectfully.

### 3.1. General self-efficacy

The Chronbach alpha of the general self-efficacy scale for before and after is *a* = 0.949 and *a* = 0.920 respectfully. In the overall sample, the mean score (*N* = 100) of the self-efficacy scale was 3.217 (±0.223) prior to training and 3.484 (±0.217) following the training. This difference is statistically significant according to Wilcoxon paired samples test (*W* = 766.00, *Z* = 5.338, *p* < 0.001). When zooming on the trainer population, the mean score for the self-efficacy scale was 3.272 (±0.13) prior to training and 3.500 (±0.01) following the training (*W* = 0, *Z* = −2.803, *p* < 0.05); while among trainees it was 3.242 (±0.10) and 3.556 (±0.08) (*W* = 0, *Z* = −2.803, *p* < 0.05) respectfully. Furthermore, differences were observed between training participants. German participants for example tended to rank their self-efficacy lower than the other participants (both before and after the training), while Turkish participants ranked their self-efficacy as highest (both before and after). The change $\left(\Delta \right)$ before and after the training in self-efficacy was highest among the Portuguese participants, whereas the Norwegian participants had mixed results in terms of self-efficacy change following the training. Although differences in participants were observed between the organization trainings, because the formats varied (full delivery of the training; only new exercises delivered; modular format), statistical significance of these differences were not computed. See complete details in Table 4.

**Table 4:** Comparison of means, and their change per item of the Self-efficacy scale between countries (*N* = 100).

Item	Germany training (*n* = 18)		Portugal training (*n* = 32)		Turkey training (*n* = 39)		Norway training (*n* = 11)	
	Mean score after training (±SD)	Mean Change^a^	Mean score after training (±SD)	Mean Change^a^	Mean score after training (±SD)	Mean Change^a^	Mean score after training (±SD)	Mean Change^a^
1. I can always manage to solve difficult problems if I try hard enough.	3.278 (±0.46)	+0.23	3.469 (±067)	+0.12	3.667 (±0.53)	+0.18	3.4 (±0.52)	+0.23
2. If someone opposes me, I can find the means and ways to get what I want.	3.056 (±0.54)	+0.39	3.313 (±0.86)	+0.29	3.667 (±0.58)	+0.52	3.200 (±0.42)	+0.12
3. It is easy for me to stick to my aims and accomplish my goals.	3.222 (±0.55)	+0.55	3.594 (±0.56)	+0.30	3.718 (±0.51)	+0.40	3.200 (±0.42)	−0.05
4. I am confident that I could deal efficiently with unexpected events.	3.278 (±0.46)	+0.23	3.594 (±0.56)	+0.27	3.692 (±0.52)	+0.42	3.400 (±0.70)	−0.02
5. Thanks to my resourcefulness, I know how to handle unforeseen situations.	3.222 (±0.55)	+0.41	3.656 (±0.55)	+0.03	3.590 (±0.59)	+0.29	3.600 (±0.70)	+0.18
6. I can solve most problems if I invest the necessary effort.	3.389 (±0.50)	+0.29	3.742 (±0.51)	+0.41	3.821 (±0.39)	+0.36	3.400 (±0.52)	+0.10
7. I can remain calm when facing difficulties because I can rely on my coping abilities.	3.167 (±0.51)	+0.26	3.719 (±0.52)	+0.42	3.718 (±0.56)	+0.40	3.500 (±0.53)	0.003
8. When I am confronted with a problem, I can usually find several solutions.	3.278 (±0.46)	+0.33	3.719 (±0.52)	+0.34	3.692 (±0.53)	+0.18	3.400 (±0.52)	+0.07
9. If I am in trouble, I can usually think of a solution.	3.167 (±0.38)	+0.31	3.75 (±0.51)	+0.39	3.744 (±0.49)	+0.26	3.600 (±0.52)	+0.27
10. I can usually handle whatever comes my way.	3.167 (±0.62)	+0.12	3.688 (±0.54)	+0.45	3.538 (±0.64)	+0.27	3.333 (±0.50)	+0.17

^a^Change in mean score was computed by subtracting the mean score prior to training from the one after the training. ^b^Scale is 1–4 for all questions.

A residual variable of the difference in self-efficacy was computed by subtracting the mean score of self-efficacy before the training from the score afterwards. Males reported higher levels of self-efficacy than women participants both before (3.275 vs. 3.218) and after the training (3.557 vs. 3.508), albeit not statistically significant (*U* = 39, *Z* = 0.79373, *p* = 0.4273) and (*U* = 39, *Z* = 0.793725, *p* = 0.4240) respectfully according to the Mann–Whitney *U*-test.

No correlation observed between age and perception of self-efficacy was observed either before (*r* = 0.087, *p* = 0.364) nor after the training (*r* = −0.034, *p* = 0.745), according to Spearman Correlation test.

### 3.2. Teamwork

The Chronbach alpha of the teamwork scale for before and after is *a* = 0.943 and *a* = 0.948 respectfully. In the overall sample, the mean score (*N* = 100) of the teamwork scale was 2.512 (±1.313) prior to training and 3.281 (±0.864) following the training. This difference is statistically significant according to Wilcoxon paired samples test (*W* = 990.0, *Z* = −5.777, *p* < 0.001). When zooming on the trainer population, the mean score for the teamwork scale was 2.636 (±0.13) prior to training and 2.643 (±0.297) following the training (*W* = 32, Z = −0.088, *p* = 0.928); while among trainees it was 2.520 (±0.11) and 3.682 (±0.28) respectfully (*W* = 0, *Z* = −2.9341, *p* < 0.05). Furthermore, differences were observed between training participants. German participants for example tended to rank their teamwork as lower than the other participants (both before and after the training), however the change $\left(\Delta \right)$ with regard to teamwork before and after the training was highest among them. Norwegian participants tended to rate their self-efficacy as highest (both before and after the training). Although differences in participants were observed between the organization trainings, because the formats varied (full delivery of the training; only new exercises delivered; modular format), statistical significance of these differences were not computed. See complete details in Table 5.

**Table 5:** Comparison of means, and their change per item of the Teamwork scale between countries (*N* = 100).

Item	Germany training (*n* = 18)		Portugal training (*n* = 32)		Turkey training (*n* = 39)		Norway training (*n* = 11)	
	Mean score after training (±SD)	Mean Change^a^	Mean score after training (±SD)	Mean Change^a^	Mean score after training (±SD)	Mean Change^a^	Mean score after training (±SD)	Mean Change^a^
1. The team leader let the team know what was expected of them through direction and command	0.333 (±0.97)	+0.29	3.625 (±0.61)	+0.49	3.538 (±0.82)	+0.07	3.909 (±0.30)	+0.49
2. The team leader maintained a global perspective	0.500 (±1.15)	+0.45	3.625 (±0.71)	+0.41	3.385 (±0.91)	+0.17	3.909 (±0.30)	+0.41
3. The team communicated effectively	2.556 (1.381)	+2.18	3.68 (±0.54)	+0.29	3.462 (±0.85)	+0.19	3.818 (±0.40)	+0.40
4. The team worked together to complete the tasks in a timely manner	2.647 (±1.498)	+2.31	3.844 (±0.45)	+0.52	3.615 (±0.67)	+0.39	3.727 (±0.47)	+0.31
5. The team acted with composure and control	2.222 (±1.396)	+1.93	3.875 (±0.34)	+0.55	3.333 (±0.74)	+0.47	3.909 (±0.30)	+0.58
6. The team morale was positive	2.111 (±1.491)	+1.77	3.875 (±0.42)	+0.44	3.667 (±0.62)	+0.28	3.909 (±0.30)	+0.33
7. The team adapted to changing situations	2.278 (±1.526)	+1.94	3.938 (±0.25)	+0.75	3.667 (±0.66)	+0.67	3.909 (±0.30)	+0.58
8. The team monitored and reassessed the situation	2.167 (±1.505)	+1.83	3.781 (±0.42)	+0.67	3.513 (±0.64)	+0.42	3.909 (±0.30)	+0.74
9. The team anticipated potential actions	1.889 (±1.568)	+1.55	3.688 (±0.47)	+0.53	3.615 (±0.67)	+0.42	3.818 (±0.40)	+0.32
10. The team prioritized tasks	2.722 (±1.364)	+2.38	3.844 (±0.45)	+0.60	3.625 (±0.54)	+0.75	3.818 (±0.40)	+0.49
11. The team followed approved standards and guidelines	2.444 (±1.294)	+2.11	3.750 (±0.44)	+0.39	3.462 (±0.68)	+0.56	3.455 (±0.52)	+0.29
12. Global rating of the team’s non-technical performance (1–10 scale)	5.278 (±2.906)	+4.51	8.656 (±1.771)	+1.44	8.795 (±1.031)	+5.82	9.091 (±0.701)	+1.76

^a^Change in mean score was computed by subtracting the mean score prior to training from the one after the training; ^b^Scale is 0–4 for questions 1–11. For question 12, scale is from 1–10.

A residual variable of the difference in teamwork was computed by subtracting the mean score of teamwork before the training from the score afterwards. Males reported higher levels of teamwork than women participants both before (2.709 vs. 2.554) and after the training (3.437 vs. 3.222). The findings were statistically significant (*U* = 11, *Z* = 3.21759 *p* < 0.001) and (*U* = 23, *Z* = 2.42961, *p* = 0.0121) respectfully, according to the Mann–Whitney *U*-test.

In addition, item 12 on the scale prompted participants to assess the global rating of the team’s non-technical performance on a scale of 1 to 10. A residual variable of the difference in responses to item 12 (global rating of the team’s non-technical performance) was computed by subtracting the mean score of this item before the training from the score afterwards. The overall mean rating was 4.43 (±3.14) prior to training and 8.22 (±2.26) following the training. This difference is statistically significant according to Wilcoxon Test (*W* = 1,605, *Z* = −5.771, *p* < 0.001). For item 12, males reported a overall higher non-technical performance of the team both before (4.681 versus 3.938) after the training (8.5 versus 7.214), albeit not significant for both prior (*U* = 958.5, *Z* = 2.42961, *p* = 0.2871) and after the training (*U* = 700.5, *Z* = 1.05842, *p* = 0.0870). No correlation observed between age and perception of teamwork was observed either before (*r* = 0.020, *p* = 0.846) nor after the training (*r* = −0.035, *p* = 0.737), according to Spearman Correlation test.

### 3.3. Quality of training

The Chronbach alpha for the quality of training index is *a* = 0.971. The quality of training was assessed once, following the training, by all participants. Trainees (*N* = 90) and trainers (*N* = 10) had two different questionnaires tailored to their perspective. Within the groups of trainees, the overall mean score of the quality of training scale was 4.345 (±0.73) and among the trainers it was 4.648 (±0.16). No differences between men and women were observed. Both types of participants assess the quality of the training as equally high. The quality of training scale is not correlated with age or the self-efficacy and teamwork scales. See Tables 6, 7.

**Table 6:** Means and percentage of top option selection per item of the quality of training questionnaire within trainees (*N* = 90).

Item	Germany trainees (*n* = 15)		Portugal trainees (*n* = 29)		Turkey trainees (*n* = 36)		Norwegian trainees (*n* = 10)	
	Mean (±SD)	% of top option	Mean (±SD)	% of top option	Mean (±SD)	% of top option	Mean (±SD)	% of top option
1. The objectives of the training were clearly defined	2.70 (±1.11)	27.7%	4.78 (±0.49)	96.2%	4.55 (±0.76)	91.0%	4.55 (±0.69)	90.0%
2. Participation and interaction were encouraged in this training	3.71 (±0.99)	67.7%	5.0 (±0.00)	100%	4.58 (±0.75)	88.8%	4.82 (±0.40)	100.0%
3. The topics covered in this training were relevant to me	2.88 (±1.05)	33.3%	4.94 (±0.25)	100%	4.37 (±0.85)	94.4%	4.91 (±0.30)	100.0%
4. The structure and contents of this training were well organized and easy to follow	3.06 (±1.19)	39.9%	4.89 (±0.34)	100%	4.47 (±0.76)	94.4%	4.73 (±0.47)	100.0%
5. The training experience will be useful in my team’s work	3.24 (±1.39)	50.0%	4.89 (±0.34)	100%	4.63 (±0.67)	94.4%	5.00 (±0.00)	100.0%
6. The trainer was well prepared and knowledgeable	3.41 (±1.33)	44.4%	4.91 (±0.39)	96.2%	4.68 (±0.66)	94.4%	4.91 (±0.30)	100.0%
7. The training objectives were met	3.00 (±1.17)	50%	4.78 (±0.42)	100%	4.61 (±0.68)	94.4%	4.91 (±0.30)	100.0%
8. The time allotted to the training was sufficient and appropriate	2.482 (±1.13)	50%	4.78 (±0.55)	93.1%	4.53 (±0.73)	91.6%	4.73 (±0.47)	100.0%
9. The logistics supporting this training were adequate	3.47 (±1.18)	61.1%	4.97 (±0.18)	100%	4.55 (±0.69)	94.4%	4.55 (±0.69)	90.0%
10. The training was appropriate to my level of experience and knowledge	3.35 (±1.22)	55.5%	4.78 (±0.75)	96.2%	4.55 (±0.72)	91.6%	4.82 (±0.40)	100.0%
11. Overall, this training was effective and useful to me	2.94 (±1.35)	44.4%	4.94 (±0.25)	100.0%	4.61 (±0.75)	94.4%	4.91 (±0.30)	100.0%

Note: ^*^% of top option indicates percentage of participants that indicate a score of 4 or 5 on a 5 point Likert scale.

**Table 7:** Means and percentage of top option selection per item of the quality of training questionnaire within trainers (*N* = 10).

	Mean (±SD)	% of top option
I have all the tools I need to train the TEAMS package	4.45 (±0.69)	90.0%
I feel competent in implementing a TEAMS training	4.54 (±0.68)	90.0%
I think my training was beneficial for the trainees	4.63 (±0.92)	90.0%
I think the training was beneficial for me as a trainer	4.82 (±0.41)	100.0%
The TEAMS training helped build trust between myself and the trainees	4.55 (±0.93)	90.0%
The trainees appreciated my training skills	4.55 (±0.93)	90.0%
I think it is important to conduct the TEAMS Training of Trainers (ToT) to improve skills and competencies of the trainers	4.91 (±0.30)	100%
I think the TEAMS training achieved its goals	4.73 (±0.47)	100%

Note: *% of top option indicates percentage of participants that indicate a score of 4 or 5 on a 5 point Likert scale. ^a^Scale is rated as 1–5 for all questions.

## 4. Discussion

When a disaster overwhelms the local disaster management system, emergency medical teams (alongside other agencies) are often requested to provide aid to the local community. Despite playing a critical role in the global health workforce, previous deployments of international medical teams have faced various obstacles and have documented shortcomings of the response (6, 7). For emergency medical teams to fortify its role and response to future events, it has been acknowledged that the development of guidelines and appropriate training is necessary to ensure the successful provision of medical services (17, 18).

Working with unacquainted team members of varying experience levels can prove challenging in a normal setting for any medical team—yet these challenges are exacerbated in austere environments with the need to adopt new responsibilities. To best prepare EMT members, trainings must focus on building capacities of an integrated multidisciplinary team (18). Multiple attempts to standardize the education and training of disaster and emergency responders have been made; however, the primary focus being the individual’s professional development rather than competencies necessary for improved operational performance. There is a need to focus educational efforts on building capacities of EMT members to adapt their competencies to work in unfamiliar, limited resource conditions and as an integrated, close-knitted multidisciplinary team (19).

The TEAMS project development stemmed from the importance of filling the gaps in facilitating operational team training for EMTs. The TEAMS 3.0 project is the follow-up project to the previously piloted training in Germany and Turkey which found overall positive attitudes of participants toward the TEAMS Training Package. This project aimed to build upon the previous training package given the unceasing evolution of both the EMC and EMT initiatives through the adding of training objectives focused on building capacities to respond to outbreaks, man-made disasters and other possible cross-border risks. Additionally, the package was delivered to four additional EMTs. The data from the project indicates that participants improved their perception of self-efficacy and team work following the training, suggesting that the training has a positive effect over those perceptual constructs among participants. These findings are aligned with the previous TEAMS pilot (15) and additional studies which indicated that high fidelity simulation based training programs contribute to enhancement of teamwork (20, 21), self-efficacy (22), and leadership competencies of medical teams (23). As these components are related to the effective performance of teams throughout deployment in disaster settings, it is expected that the training package will improve the operational preparedness of EMTs.

Previous findings from training programs indicated the gender is often correlated with teamwork levels or self-efficacy, where men often exhibited higher self-efficacy, while women higher levels of team work (24–26). In contrast to previous work, gender was not found to correlate with self-efficacy or teamwork prior or following the training program.

Finally, the adapted and updated TEAMS training package appears to be similarly a high-quality product, which was considered by its users to be a useful and appropriate tool for addressing their perceived needs. While in all four trainings the assessment parameters improved following the delivery of the training package, differences were observed per each country. For example, the results suggest that German participants were more critical toward the TEAMS training compared to the other participants. It is possible that this is resulting from the format of the German training, which was conducted modularly in tandem to additional external trainings of the organization. The differences observed among all four EMTs may be resulting from the diversity of their cultural characteristics, type and size of EMT, mix of personnel, and experience in previous deployments to disasters, or resulting from the way in which the training package was delivered (in full, modularly, only three new exercises). Despite these differences, as improvements were observed among all settings, this suggests that the training programs may be beneficial among various EMTs and in varying formats that can be adapted to needs of the respective EMT. The TEAMS training may need to be adapted into the cultural and sociological context of each country prior to the implementation of the training, which may further enhance the results. The complete package including the newly developed exercises are available online, free-of-charge, to any relevant stakeholder interested in implementing it in the local EMT context. By creating a validated, cost-effective training tool for EMTs, TEAMS project further contributes to the global effort to promote a higher quality EMT system through providing a more standardized training protocol that may ultimately benefit the way in which health delivery is provisioned to affected populations, in line with the EMT WHO vision.

### 4.1. Limitations

The study has several limitations which must be considered. First, the study employs a questionnaire that might be subject to reporting-bias due to the fact that it measures construct through reporting, rather than objective assessment. Second, the study was performed among EMTs, primarily in Europe. Though it stands to reason that the conclusions may be generalized and applicable to other EMT organizations in different societies, this should be made with caution, preferably following further study to evaluate the applicability of the findings in additional EMT organizations outside of Europe. Third, for practical considerations, the tool used assessed only the construct reported and cannot control for possible confounders associated with these constructs. Additionally, several factors must be taken into account when considering the findings of the study including but not limited to the language in which the evaluation tool was administered (English, as a non-native language for three of the participating EMTs, while in Turkey, Turkish was utilized); differences resulting from heterogeneity (e.g., professional status/seniority levels etc.) of groups in the respective countries; as well as differences in training contexts between the varied countries.

## Data availability statement

The raw data supporting the conclusions of this article will be made available by the authors, without undue reservation.

## Ethics statement

The studies involving human participants were reviewed and approved by Sheba Medical Center (number SMC-9777-22 from October 2, 2022). The patients/participants provided their written informed consent to participate in this study.

## Author contributions

AK and MB conceived the study, provided statistical advice on study design, and analyzed the data. NC, MB, KP, and LR supervised the conduct of the trial and data collection. AK undertook recruitment of participating centers and patients and managed the data, including quality control. NC chaired the data oversight committee. AK drafted the manuscript and takes responsibility for the paper as a whole. All authors contributed substantially to its revision.

## Funding

This study was performed in the context of the European Commission funded project ‘TEAMS 3.0.’ The authors acknowledge the financial support from Directorate-General for European Civil Protection and Humanitarian Aid Operations (Grant Number: 101004935).

## Conflict of interest

The authors declare that the research was conducted in the absence of any commercial or financial relationships that could be construed as a potential conflict of interest.

## Publisher’s note

All claims expressed in this article are solely those of the authors and do not necessarily represent those of their affiliated organizations, or those of the publisher, the editors and the reviewers. Any product that may be evaluated in this article, or claim that may be made by its manufacturer, is not guaranteed or endorsed by the publisher.
