# Supplementary material for: Enhancing disaster response of emergency medical teams through “TEAMS 3.0” training package—Does the multidisciplinary intervention make a difference?
Source: Front Public Health. 2023 Apr 12;11:1150030. doi: 10.3389/fpubh.2023.1150030 (PMC10130359; doi:10.3389/fpubh.2023.1150030)
Supplement: Supplementary file 1 [file Data_Sheet_1.docx]

**ANNEX 1 - Questionnaire**

General Self-Efficacy Scale

Dear participant,

Please indicate the following information:

Gender: Male / Female / Other

Role in team: Trainer / Trainee

Year of birth: ______________

Serial number of participant (last four digit of your cellular phone): ____________________

Type of evaluation: BEFORE / AFTER

Please select the appropriate response for each item below on a scale of 1 to 4 where 1 means "Not at all true" and 4 means "Exactly true".

| # | Item | 1 Not at all true | 2 Hardly true | 3 Moderately true | 4  Exactly true |
| --- | --- | --- | --- | --- | --- |
|  | I can always manage to solve difficult problems if I try hard enough. | 1 | 2 | 3 | 4 |
|  | If someone opposes me, I can find the means and ways to get what I want. | 1 | 2 | 3 | 4 |
|  | It is easy for me to stick to my aims and accomplish my goals. | 1 | 2 | 3 | 4 |
|  | I am confident that I could deal efficiently with unexpected events. | 1 | 2 | 3 | 4 |
|  | Thanks to my resourcefulness, I know how to handle unforeseen situations. | 1 | 2 | 3 | 4 |
|  | I can solve most problems if I invest the necessary effort. | 1 | 2 | 3 | 4 |
|  | I can remain calm when facing difficulties because I can rely on my coping abilities. | 1 | 2 | 3 | 4 |
|  | When I am confronted with a problem, I can usually find several solutions. | 1 | 2 | 3 | 4 |
|  | If I am in trouble, I can usually think of a solution. | 1 | 2 | 3 | 4 |
|  | I can usually handle whatever comes my way. | 1 | 2 | 3 | 4 |

Teamwork


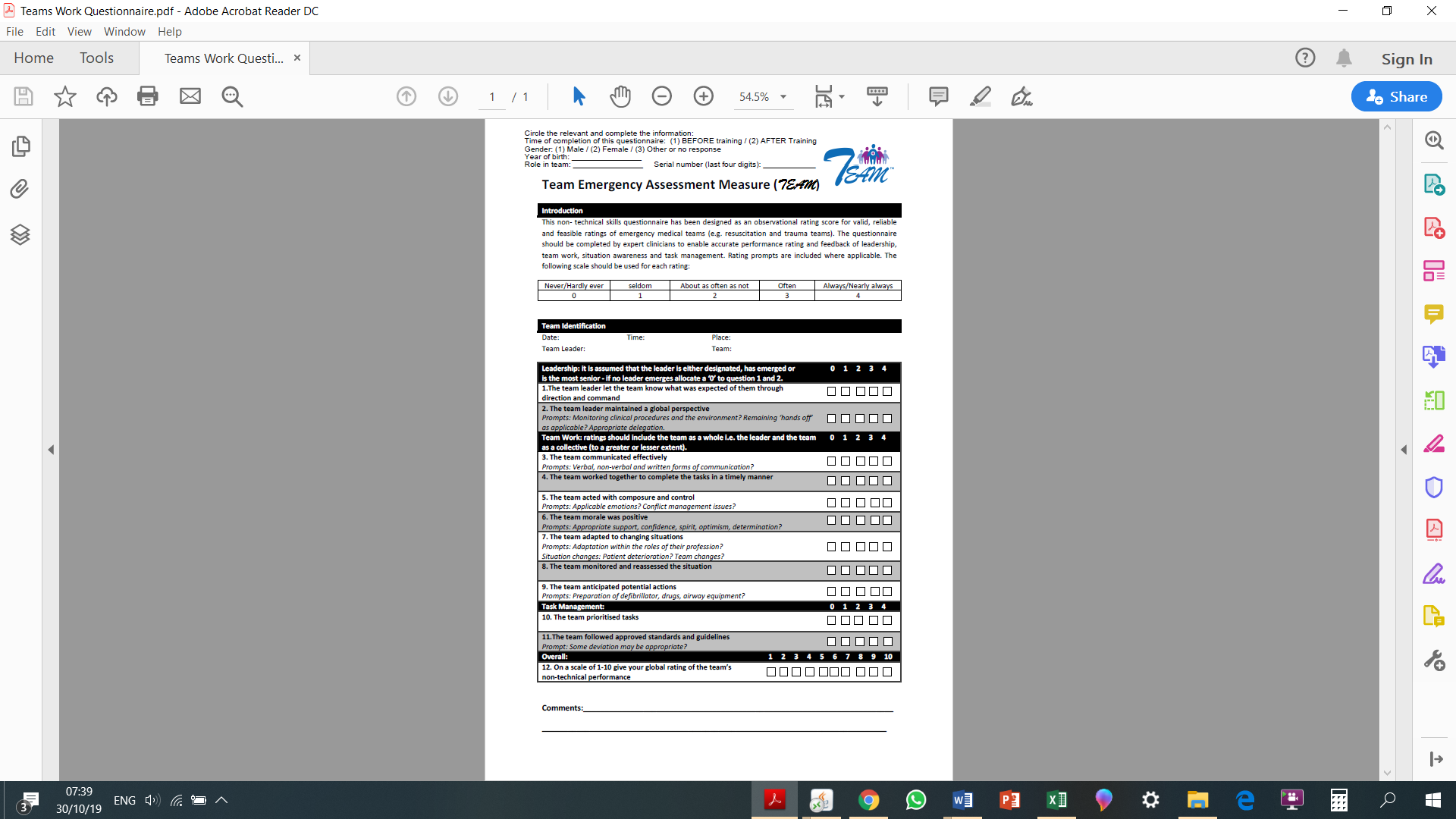


Quality of Training Questionnaire (for trainees)

Dear participant,

Please indicate the following information:

Gender: Male / Female / Other

Year of birth: ______________

Serial number of participant (last four digit of your cellular phone): ____________________

Please respond to these items assessing **your perception of the quality** of your recent training. Rate each of the following statements by circling the appropriate number on a scale of 1 to 5 where 1 means you strongly disagree and 5 means you strongly agree.

|  | Strongly  Disagree | Somewhat Disagree | Neither Agree  Nor Disagree | Somewhat Agree | Strongly Agree |
| --- | --- | --- | --- | --- | --- |
| 1. The objectives of the training were clearly defined | 1 | 2 | 3 | 4 | 5 |
| 2. Participation and interaction were encouraged in this training | 1 | 2 | 3 | 4 | 5 |
| 3. The topics covered in this training were relevant to me | 1 | 2 | 3 | 4 | 5 |
| 4. The structure and contents of this training were well organized and easy to follow | 1 | 2 | 3 | 4 | 5 |
| 5. The training experience will be useful in my team's work | 1 | 2 | 3 | 4 | 5 |
| 6. The trainer was well prepared and knowledgeable | 1 | 2 | 3 | 4 | 5 |
| 7. The training objectives were met | 1 | 2 | 3 | 4 | 5 |
| 8. The time allotted to the training was sufficient and appropriate | 1 | 2 | 3 | 4 | 5 |
| 1. The logistics supporting this training were adequate | 1 | 2 | 3 | 4 | 5 |
| 1. The training was appropriate to my level of experience and knowledge | 1 | 2 | 3 | 4 | 5 |
| 1. Overall, this training was effective and useful to me | 1 | 2 | 3 | 4 | 5 |

Additional comments? ______________________________________________________________________________________

Quality of Training Questionnaire (for trainers)

Dear trainer,

Please indicate the following information:

Gender: Male / Female / Other

Year of birth: ______________

Serial number of participant (last four digit of your cellular phone): ____________________

Please respond to these items assessing **your perception of the quality** of your recent training. Rate each of the following statements by circling the appropriate number on a scale of 1 to 5 where 1 means you strongly disagree and 5 means you strongly agree.

|  | Strongly  Disagree | Somewhat Disagree | Neither Agree  Nor Disagree | Somewhat Agree | Strongly Agree |
| --- | --- | --- | --- | --- | --- |
| 1. I have all the tools I need to train the TEAMS package | 1 | 2 | 3 | 4 | 5 |
| 1. I feel competent in implementing a TEAMS training | 1 | 2 | 3 | 4 | 5 |
| 1. I think my training was beneficial for the trainees | 1 | 2 | 3 | 4 | 5 |
| 1. I think the training was beneficial for me as a trainer | 1 | 2 | 3 | 4 | 5 |
| 1. The TEAMS training helped build trust between myself and the trainees | 1 | 2 | 3 | 4 | 5 |
| 1. The trainees appreciated my training skills | 1 | 2 | 3 | 4 | 5 |
| 1. I think it is important to conduct the TEAMS Training of Trainers (ToT) to improve skills and competencies of the trainers | 1 | 2 | 3 | 4 | 5 |
| 1. I think the TEAMS training achieved its goals | 1 | 2 | 3 | 4 | 5 |

Tell us about your experience as a trainer: in what ways can the TEAMS Training of Trainers process improve? What worked really well and which aspects were you not satisfied with?

______________________________________________________________________________________
